# Assessment of scattered and leakage radiation from ultra-portable X-ray systems in chest imaging: An independent study

**DOI:** 10.1371/journal.pgph.0003986

**Published:** 2025-01-24

**Authors:** Leonie E. Paulis, Roald S. Schnerr, Jarred Halton, Zhi Zhen Qin, Arlene Chua

**Affiliations:** 1 Médecins Sans Frontières, International, Amsterdam, The Netherlands; 2 Department of Digital Health, Stop TB, Geneva, Switzerland; 3 Department of Medical Physics, Maxima Medical Center, Veldhoven, The Netherlands; 4 Department of Radiology and Nuclear Medicine, Maastricht University Medical Center, Maastricht, The Netherlands; 5 Médecins Sans Frontières, International, Geneva, Switzerland; University of California San Francisco, UNITED STATES OF AMERICA

## Abstract

Ultraportable (UP) X-ray devices are ideal to use in community-based settings, particularly for chest X-ray (CXR) screening of tuberculosis (TB). Unfortunately, there is insufficient guidance on the radiation safety of these devices. This study aims to determine the radiation dose by UP X-ray devices to both the public and radiographers compared to international dose limits. Radiation dose measurements were performed with four UP X-ray devices that met international criteria, utilizing a clinically representative CXR set-up made with a thorax phantom. Scatter and leakage radiation dose were measured at various positions surrounding the phantom and X-ray tube, respectively. These measurements were used to calculate yearly radiation doses for different scenarios based on the median of all UP X-ray devices. From the yearly scatter doses, the minimum distances from the phantom needed to stay below the international public dose limit (1 mSv/year) were calculated. This distance was longest in the direction back towards the X-ray tube and shortest to the left/right sides of the phantom, e.g., 4.5 m and 2.5 m resp. when performing 50 exams/day, at 90 kV, 2.5 mAs and source skin distance (SSD) 1 m. Additional calculations including leakage radiation were conducted at a typical radiographer position (i.e., behind the X-ray tube), with a correction factor for wearing a lead apron. At 2 m behind the X-ray tube, a radiographer wearing a lead apron could perform 106 exams/day at 2.5 mAs and 29 exams/day at 10 mAs (90 kV, SSD 1 m), while keeping his/her radiation dose below the public dose limit (1 mSv/year) and well below the radiographer dose limit (20 mSv/year). In most CXR screening scenarios, the radiation dose of UP X-ray devices can be kept below 1 mSv/year by employing basic radiation safety rules on time, distance and shielding and using appropriate CXR exposure parameters.

## Introduction

Recent advancements in radiological equipment have led to the development of UP X-ray devices, which are X-ray systems that are designed to fit within a suitcase or backpack and can be moved regularly to areas of need [1,2]. These systems are composed of a low-weight, battery-powered X-ray tube combined with a highly sensitive and dose-efficient digital detector, and can be used for a range of general X-ray examinations, such as chest and extremities [2]. One main advantage of these systems, besides their portability, is their ability to be used in settings where conventional X-ray infrastructure requirements (e.g., stable power supply) are not available. Consequently, these systems have the potential to increase access to X-ray facilities for communities in low- and middle-income countries (LMIC), where the availability of functioning X-ray equipment is limited [3,4].

UP X-ray devices have proven particularly useful when they can be paired with artificial intelligence (AI) powered computer-aided detection (CAD) software. This is because CAD tools provide automated and standardized image interpretation without the need of human interpretation by expert readers, which are scarce in LMIC [5]. Currently, the main application of UP X-ray devices with CAD tools, and the only use case recommended by the World Health Organization (WHO) [6], is CXR screening for pulmonary TB [5,7–10]. TB has the highest annual mortality of all infectious diseases, despite being treatable [3]. However, for adequate treatment early diagnosis is critical, which can be achieved by systematic and large-scale CXR screening in high-risk subpopulations to triage patients for scarce and expensive molecular tests [11,12]. UP X-ray devices with CAD are an ideal candidate to provide this CXR screening, because TB is concentrated in LMIC.

Recent studies have shed light on the challenges faced when using UP X-ray devices for CXR screening, with a main concern being the absence of radiation safety guidelines [5]. Existing local regulatory standards in LMIC are often primarily designed for fixed X-ray machines in a dedicated hospital room with lead barriers [13]. Applying the same guidelines to UP X-ray devices restricts their potential to be regularly moved from location to location, and be used in resource-limited conditions, which is their primary intended use [1]. Therefore, it is important to also develop guidelines for safe use of UP X-ray devices, to minimize radiation related health risks.

**Fig 1:**
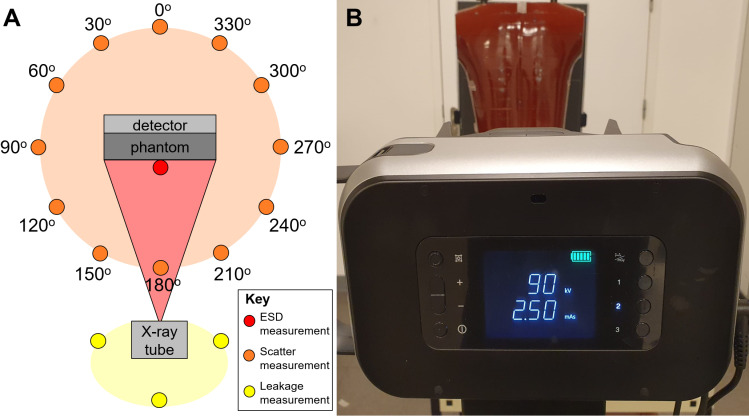
Experimental set-up. A. Schematic top view of the experimental set-up with in light red the primary X-ray beam, in light orange the scattered radiation field originating from the phantom and in light yellow the leakage radiation field emanating from the X-ray tube. Entrance skin dose (ESD) measurements were performed at the posterior side (in front) of the phantom. Scattered radiation measurements were performed at 1 m from the center of the phantom at 30° intervals. Leakage radiation measurements were performed at 0.5 m from the X-ray tube to the left, right and back. B. The experimental set-up seen from the position of the radiographer, showing the X-ray tube, phantom and detector.

From a radiation safety perspective, the health risk to the individual patient by a CXR exam is negligible due to the low X-ray radiation dose needed (equivalent to a few days of natural background radiation) [14]. Particular focus, though, should be on the radiation safety of radiographers and others (the ‘public’) that are in the close vicinity of UP X-ray devices during their operation, including non-radiographer staff and attendees at screening sites, especially given the community-based use case. Although they are not exposed to the primary X-ray beam (which is focused on the patient), they are exposed to scattered and leakage radiation (S1 Table, Fig 1A). Scattered radiation is X-ray radiation that, upon interaction of the primary X-ray beam with the patient, is scattered from the patient to its surroundings. The scattered radiation dose depends on the patient’s exposed body part and the primary radiation dose used (which is determined by scan parameters, such as kV, mAs and SSD (Table 1)). For example, an adult CXR requires a higher primary radiation dose than a child CXR and therefore produces more scattered radiation. Leakage radiation is X-ray radiation that emanates from the X-ray tube’s protective housing in other directions than the primary X-ray beam during the exposure [15]. Scattered and, especially, leakage radiation are much lower in intensity than the primary X-ray beam [15], however, a high patient throughput, i.e., in a CXR screening setting, may still result in a substantial cumulative (total) radiation dose for people working there (e.g., radiographers or other program staff) as opposed to people that spend only limited time at a CXR screening site (e.g., people in waiting rooms).

**Table 1:** Description of X-ray scan parameters and radiation protection measures in relation to patient, scattered and leakage radiation dose [17].

Parameter	Description	Relation with:		
		Patient dose	Scatter dose	Leakage dose
Tube voltage (kV)*	Affects maximal energy & number of X-ray photons	Non-linear increase with increasing kV	**	**
Tube current • exposure time (mAs)	Affects number of X-ray photons	Linear increase with increasing mAs	**	**
Source skin distance (SSD)	Distance X-ray tube to patient	Inverse quadratic decrease with increasing SSD	**	None
Time	Time spent in X-ray field	None	Linear decrease with decreasing time	***
Distance	Distance to X-ray source	None	Inverse quadratic decrease with increasing distance	***
Shielding	Material that absorbs X-ray photons	None	Non-linear decrease with increasing thickness	***

*For UP X-ray devices, the WHO/IAEA criteria require the maximum kV to be at least 90 kV.

**See patient dose.

***See scatter dose

To minimize radiation health risks, fundamental radiation safety measures based on time, distance and shielding are crucial (Table 1). In a conventional X-ray department, walls are typically covered with lead (known for its shielding capacity). For an UP X-ray set-up, however, this is not an option and transporting lead barriers is not feasible. Instead, one should minimize the time spent close to the set-up during its use, maximize his/her distance to the set-up and use protective clothing (e.g., a lead apron and thyroid collar). Regardless of the set-up and radiation protection measures in place, radiation exposure levels are subjected to international radiation dose limits of an effective dose of 1 mSv/year to the public and up to 20 mSv/year for radiographers (S2 and S3 Tables) [13,16]. These limits ensure radiation doses are too low to cause short-term health effects (e.g., red skin) and have a minimal risk of long-term effects (i.e., cancer or genetic defects) [14]. Importantly, international guidelines require individual (personal) radiation dose monitoring to be provided to radiographers when they are expected to receive more than 1 mSv/year, to guarantee their dose will be below 20 mSv/year [16].

To gain an insight in the radiation safety of UP X-ray devices, with particular focus on CXR screening, the current study conducted an independent analysis of both the scattered and leakage radiation from CXR exams by all four commercially available UP X-ray systems that met the criteria from the WHO and the International Atomic Energy Agency (IAEA) at the time of the study [1]. The objective of this study is to contribute to guidance on the safe use of UP X-ray devices in areas outside dedicated X-ray departments, while complying with internationally recognized standards [13,16].

## Methods

The study protocol consisted of 2 steps:

1. Measurements of the radiation doses from CXR exams of a thorax radiation phantom, including: 1) ESD, as a measure of UP X-ray tube output; 2) scattered radiation at multiple positions around the phantom; 3) leakage radiation close to the X-ray tube.
2. Calculations of the yearly radiation doses due to scattered radiation, leakage radiation, and the combined dose, comprising both scattered and leakage radiation, to assess the cumulative consequence of routine radiation exposure.

### Experimental set-up

For the radiation dose measurements, four UP X-ray devices were selected that met the WHO/IAEA criteria at the time of the study (September – December 2022). Device characteristics are shown in Table 2.

**Table 2:** UP X-ray device characteristics.

System*	Range		Recommended settings for CXR		
	kV	mAs	kV	mAs	SSD [m]
Fuji	50–90	0.20–2.50	90	0.5	0.8
MinXray	40–90	0.2–10.0	90	1.0	1.6
Sinopharm	40–100	0.4–50.0	90	2.5	1.3
Delft Imaging	40–90	0.1–10.0	90	1.2	1.3

*Fuji: FDR Xair (Fuji Film, Japan), MinXray: Impact Wireless (MinXray, USA), Sinopharm: SR-1000 (Shantou Institute of Ultrasonic Instruments Co. Ltd. and Sinopharm Biotech, China), Delft Imaging: Delft Light (Delft Imaging, Netherlands).

The study was conducted at the Department of Radiology and Nuclear Medicine, Maastricht University Medical Center (the Netherlands). No approval of the Institutional Review Board was needed, because the study did not involve human subjects. The UP X-ray devices were positioned in a clinically representative setup for CXR with an anthropomorphic thorax radiation phantom (Alderson phantom (Radiology Support Devices Inc.,USA)) positioned at chest height (1.4 m) in posterior-anterior (PA) orientation with respect to the X-ray tube and the X-ray detector positioned at its anterior side (Fig 1B). This phantom’s composition is representative of a human thorax, having similar interaction with X-ray radiation and thus creating a comparable level of scattered radiation. The X-ray beam was collimated to the phantom’s thorax. Basic quality control of the primary X-ray beam was performed prior to the measurements.

### Radiation dose measurements

Radiation dose measurements were performed as described below using a Piranha Multi dosimeter version 5.7 (RTI group, Sweden) with the external dose probe connected (air kerma dose range 0.1 nGy–1.5 kGy, air kerma accuracy 5%). When applicable, the radiation dose was converted from Gy to Sv using a conversion factor of 1.4 (S2 Table) [18]. All results were anonymized with respect to the individual X-ray systems.

### Entrance skin dose (ESD)

To determine the X-ray radiation output of the UP X-ray devices for a single CXR exam, the ESD was measured with the dosimeter on the posterior side of the phantom in the center of the field of view (where the X-ray beam entered the phantom) (Fig 1A; bright red dot). ESD was measured at 90 kV for 0.5, 1.0 and 2.5 mAs (encompassing the manufacturer’s recommendations) and the maximum mAs of each individual system (highest dose scenario). The ESD from Sinopharm at maximum mAs (50 mAs) was interpolated to the ESD at 10 mAs to preserve anonymity. Measurements were performed at SSD 1 m and 1.8 m, to encompass the range recommended by the American College of Radiology [19].

### Scattered radiation dose

To characterize the scattered radiation dose pattern as a function of the angle with the phantom, dose measurements were performed with the dosimeter at chest height at 1 m from the center of the phantom at 30° increments (Fig 1A; bright orange dots). CXR exams were made with scan parameters 90 kV and both 2.5 mAs and maximum mAs, for an SSD of 1 m and 1.8 m. The setting of 2.5 mAs was selected as an indicative mAs at the upper limit of the normal range of mAs values recommended by manufacturers for clinical use, whereas maximum mAs was selected to illustrate the highest dose scenario.

### Leakage radiation dose

Leakage radiation dose was measured according to IEC 60601: behind and to the left and right of the X-ray tube (Fig 1A; bright yellow dots). The collimator was closed and blocked with 5 cm thick lead blocks. Measurements were performed with the dosimeter at 0.5 m from the X-ray anode at 90 kV and maximum mAs for the system, with a limit of 10 mAs. The leakage dose at 0.5 m was converted to the leakage dose at 1 m using the inverse square law:


$$\frac{D_{L,0.5m}}{D_{L,1m}}=\frac{{\left(1m\right)}^{2}}{{\left(0.5m\right)}^{2}},$$

with D_L,0.5m_ = measured leakage dose per exam at 0.5 m from the X-ray tube [Gy] and D_L,1m_ = calculated leakage dose per exam at 1 m from the X-ray tube [Gy].

For each system, the maximum leakage dose of the three measurements was compared to international dose limit for leakage radiation (1 mGy/h).

### Calculation of yearly radiation dose

Yearly radiation doses were calculated at specified positions relative to the UP X-ray set-up, as described below. This means that only an individual standing at a certain position the entire year will receive the calculated radiation dose. Depending on whether an individual is a member of the public, including non-radiographer program staff, or a radiographer, his/her radiation dose is subjected to the international limit of 1 mSv/year or 20 mSv/year, respectively [13,16]. In the analysis, the public dose limit (1 mSv/year) was used as a cut-off, irrespective of whether individuals belong to the public or radiographers.

### Scattered radiation dose

The yearly scatter dose at 1 m from the phantom was calculated at each angle from the scatter dose measurements of a single CXR exam at 90 kV and both 2.5 mAs and 10 mAs, for an SSD of 1 m and 1.8 m. For 2.5 mAs the scatter measurements of all four systems were used. For 10 mAs, only the scatter measurements of MinXray, Sinopharm and Delft Imaging were used, because Fuji is limited to 2.5 mAs. The scatter dose of Sinopharm at 50 mAs was interpolated to the scatter dose at 10 mAs.

Yearly scatter dose was calculated for a workload of 50, 100 and 200 exams per day, by multiplying, for each angle, the median value of the systems with the total number of exams per year (assuming 5 days/week, 52 weeks/year):


$$D_{S\_\text{year},1m}=1.4\;\cdot \;{\overset{⌣}{D}}_{S,1m}\cdot \;W\cdot \;5\;\cdot 52\;,$$

with D_S_year,1m_ = calculated yearly scatter dose at 1 m from the phantom [Sv], Ď_S,1m_ = median measured scatter dose per exam at 1 m from the phantom [Gy], W = workload [exams/day], 1.4 = conversion factor Gy to Sv [18].

Subsequently, for each angle, the distance from the center of the phantom was calculated at which the yearly scatter dose was 1 mSv/year (r_1mSv_) [13,16]. This was done using the inverse square law:


$$\frac{D_{S\_year,1m}}{1mSv}=\frac{{r_{1mSv}}^{2}}{{\left(1m\right)}^{2}}$$

From this, 1 mSv/year isodose lines were plotted that showed for each angle the minimum distance to the phantom to stay below the public radiation dose limit.

### Leakage radiation dose

To calculate the yearly leakage dose at 1 m from the X-ray tube, the maximal leakage dose of the three measurements per system at 90 kV was used. For Fuji, the leakage dose measured at 2.5 mAs was used, whereas for MinXray, Sinopharm and Delft Imaging the leakage dose at 2.5 mAs was calculated from the leakage dose measured at 10 mAs.

The yearly leakage dose was calculated for a workload of 50, 100 and 200 exams per day, by multiplying the median value of the maximum dose per system at 2.5 mAs with the total number of exams per year (assuming 5 days/week, 52 weeks/year):


$$D_{L\_\;\text{year},1m}=\;1.4\cdot {\overset{⌣}{D}}_{L,1m}\cdot W\cdot 5\;\cdot 52\;,\;$$

with D_L_year,1m_ = calculated yearly leakage dose at 1 m from the X-ray tube [Sv], Ď_L,1m_ = median calculated leakage dose per exam at 1 m from the X-ray tube [Gy], W = workload [exams/day], 1.4 = conversion factor Gy to Sv [18].

### Combined radiation dose behind the X-ray tube

At the position behind the X-ray tube not only scattered, but also leakage radiation contribute to the combined yearly radiation dose. This is because leakage radiation is only significant close to the X-ray tube, due to its very low intensity [15]. This position is typically only occupied by the radiographer, whose distance to the X-ray tube is constrained by the length of the exposure cord, which is connected to the X-ray tube to operate the UP X-ray device.

The combined radiation dose was determined at a distance of 1 m and 2 m behind the X-ray tube for 90 kV and 1 mAs, 2.5 mAs and 10 mAs, for both SSD 1 m and 1.8 m. For each scenario, the scatter and leakage doses of a single exam were calculated at these positions. For this, for scattered radiation, the median scatter dose measurements at 2.5 mAs and 10 mAs at 1 m from the phantom (Ď_S,1m_) in the direction of the X-ray tube (180°) were used. For 1 mAs (which was not measured), linear extrapolation of the median dose at 2.5 mAs was used to obtain the dose at 1 mAs. Next, these median doses at 1, 2.5 and 10 mAs were converted from the dose at 1 m from the phantom (Ď_S,1m_) to the dose at 1 m or 2 m from the X-ray tube (D_S,r_tube_) using inverse square law:


$$\frac{D_{S,1m}}{D_{S,r_{tube}}}=\frac{{\left(r_{phantom\_radiographer}\right)}^{2}}{{\left(1m\right)}^{2}},$$

with Ď_S,1m_ as defined above, D_S,r_tube_ = calculated scatter dose per exam at distance ‘r_tube_’ from the X-ray tube [Gy], r_phantom_radiographer_ = SSD + r_tube_ [m], with r_tube_ = 1 m or 2 m.

For leakage radiation, for 2.5 mAs the median leakage dose per exam of the four systems at 1 m from the X-ray tube (Ď_L,1m_) was used. The doses at 1 mAs and 10 mAs, were obtained by interpolating the dose at 2.5 mAs. To calculate the doses at 2 m from the X-ray tube, the doses at 1 m were converted using inverse square law:


$$\frac{D_{L,1m}}{D_{L,2m}}=\frac{{\left(2m\right)}^{2}}{{\left(1m\right)}^{2}}$$

with Ď_L,1m_ as defined above, and D_L,2m_ = calculated leakage dose per exam at 2 m from the X-ray tube [Gy].

Subsequently, for each scenario, the yearly scatter and leakage doses at 1 m and 2 m behind the X-ray tube were calculated from the scatter and leakage dose per exam for a range of workloads (0 to >1000 exams per day) as described previously (assuming 5 days/week, 52 weeks/year). The combined (total) dose at 1 m and 2 m behind the X-ray tube (D_radiographer_year_) was defined as the sum of the yearly scatter and leakage doses:


$$D_{\text{radiographer}\_\text{year}}=D_{S\_\text{year}}+D_{L\_\text{year}},$$

From these data, the number of exams per day was determined at which the combined dose exceeded the international public dose limit (1 mSv/year). Additional calculations were performed to correct for wearing a 0.25 mm single layer lead equivalent protective apron and thyroid collar. For this, both the scatter and leakage dose per exam were converted with a correction factor of 5 [20].

## Results

### Entrance skin dose

**Fig 2:**
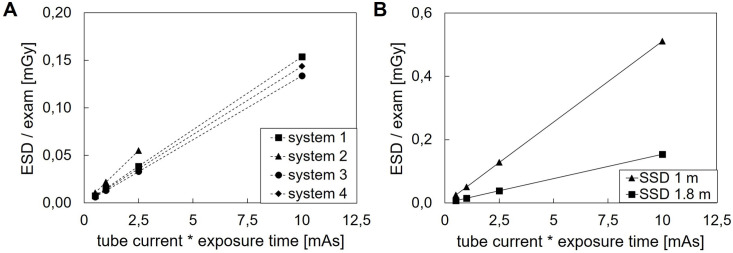
ESD of a single PA CXR phantom exam. A. ESD at 90 kV and SSD 1.8 m for all systems. B. ESD of system 1 (the median ESD of all systems) at 90 kV for SSD 1 m and 1.8 m.

The ESD of a single PA CXR exam is shown in Fig 2. Fig 2A shows the ESD of the individual UP X-ray systems for 0.5–10 mAs (SSD 1.8 m, 90 kV). The ESD increased linearly with increasing mAs (r^2^ = 1 for each system), which is in accordance with literature [17]. The effect of SSD on ESD is shown in Fig 2B. The ESD decreased with increasing SSD, following the inverse square law [17], with a deviation of max 10%, caused by inherent inaccuracy of the dosimeter (5%) and experimental set-up.

### Scattered radiation dose

**Fig 3:**
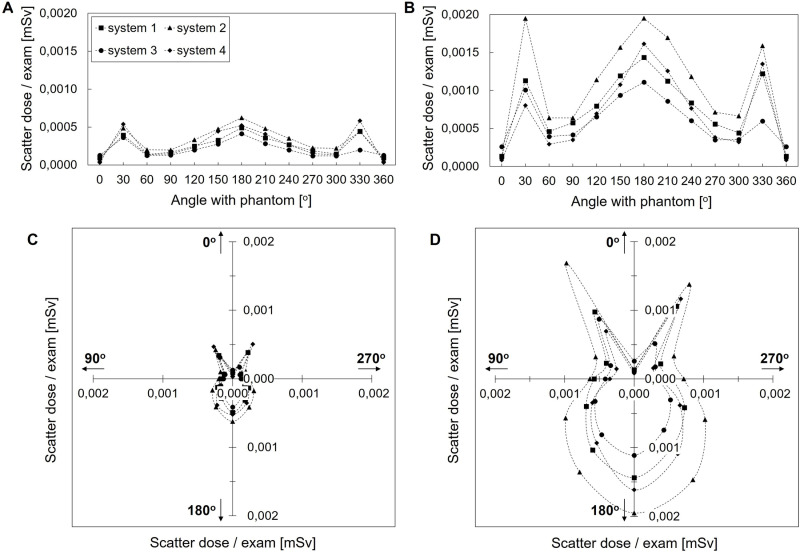
Scatter dose per exam at 1 m from Alderson phantom for all systems. A & C. Scatter dose pattern at 90 kV, 2.5 mAs and SSD 1.8 m. B & D. Scatter dose pattern at 90 kV, 2.5 mAs and SSD 1 m. In C & D the arrows indicate the angular distribution, which is identical to Fig 1A, with each point representing a 30^0^ increment counter clockwise from 0° to 360° (the upper y-axis).

The scattered radiation dose pattern from a single PA CXR exam at 1 m from the Alderson phantom is shown in Fig 3 (90 kV, 2.5 mAs, SSD 1.8 m and 1 m). Differences in scatter dose between UP X-ray systems were relatively small. For all systems, highest scatter dose was observed in the direction back towards the X-ray tube (180°; *i.e.,* backscatter) and behind the detector’s edges (30° and 330°), both positions where X-rays encounter little attenuation by tissue before they are reflected out of the phantom. At these positions the scatter dose ranged from 0.2–0.6 µSv for SSD 1.8 m and 0.6–2.0 µSv for SSD 1 m. The lowest scatter dose was observed behind the center of the detector (0°) and perpendicular to the X-ray primary beam (270–300° and 60–90°), because of higher absorption by the larger tissue mass that has to be traversed and the inherent physics of scatter as described by the Klein-Nishina formula [21]. At these positions, the scatter dose ranged from 0.0–0.2 µSv for SSD 1.8 m and 0.1–0.7 µSv for SSD 1 m. The scatter dose was lower for SSD 1.8 m compared to SSD 1 m (Fig 3A and 3C vs. Fig 3B and 3D) and increased linearly with increasing mAs from 2.5 mAs to 10 mAs.

**Fig 4:**
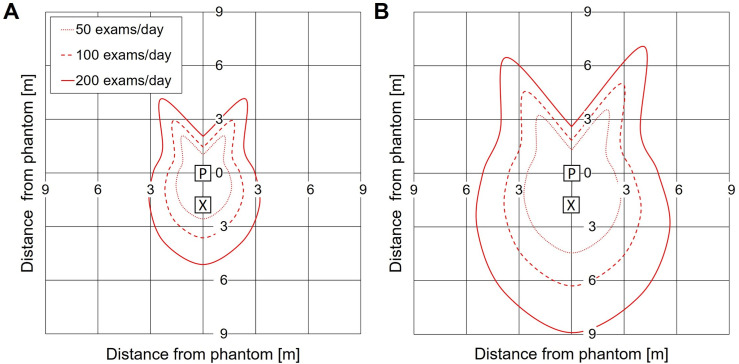
1 mSv/year isodose ‘bunnies’ for various workloads. A and B. Minimum distance to the center of the phantom for 90 kV, 2.5 mAs and SSD 1.8 m and 1 m, resp. at which the yearly scattered radiation dose was below 1 mSv/year. P and X indicate position of the phantom and X-ray tube. The angular distribution is identical to Fig 1A.

In Fig 4 the 1 mSv/year isodose lines are shown that correspond to the distance from the center of the Alderson phantom for each angular position (0°–360°) at which the yearly scatter dose exceeded the international public radiation dose limit (at 90 kV, 2.5 mAs, for SSD 1.8 m and 1 m) [13,16]. The isodose lines enclosed a ‘bunny’ shape around the phantom: The longest distance (corresponding to the highest scatter dose measured at 1 m) was observed in the direction back towards the X-ray tube (180°, the bunny’s chin) and behind the edges of the detector (30° and 330°_,_ the bunny’s ears). The shortest distance (corresponding to the lowest scatter dose measured at 1 m) was observed behind the center of the detector (0°) and to the left and right of the phantom (60–90° and 270–300°_,_ the bunny’s cheeks).

At the position with highest scatter dose (*i.e.,* back towards the X-ray tube (180°)), a workload of 50 exams/day required a distance of 2.6 m (SSD 1.8 m) and 4.5 m (SSD 1 m) from the center of the Alderson phantom to remain below the public dose limit of 1 mSv/year. Increasing the workload to 200 exams/day, increased the 1 mSv/year-distance to 5.1 m for SSD 1.8 m and 8.9 m for SSD 1 m. At the position with lowest scatter dose (*i.e.,* to the left and right of the phantom (60–90° and 270–300°), a workload of 50 exams/day resulted in a 1 mSv/year-distance from the center of the Alderson phantom of 1.5 m for SSD 1.8 m and 2.5 m for SSD 1 m. Increasing the workload to 200 exams/day, increased the 1 mSv/year-distance to 3.0 m for SSD 1.8 m and 5.1 m for SSD 1 m.

### Leakage radiation dose

For all systems, the leakage radiation dose rate of a single CXR exam was below the international limit of 1 mGy/h. The leakage dose of a single CXR exam (90 kV; 2.5 mAs) at 1 m from the X-ray tube was only minor (0–0.1 µGy) (Table 3). However, the yearly cumulative leakage dose at 1 m was substantial, ranging from 0.6 to 2.4 mSv/year for 50 to 200 exams/day. Leakage radiation is independent from SSD.

**Table 3:** Leakage of primary radiation from the X-ray tube (90 kV, 2.5 mAs, 1 m from X-ray tube).

	Leakage radiation per exam [µGy]		
	Position vs. X-ray tube		
	Left	Right	Behind
**System 1**	0.013	0.030	0.031
**System 2**	0.038	0.116	0*
**System 3**	0.014	0.011	0*
**System 4**	0.022	0.034	0.025

*Below the detection limit of the dosimeter.

### Combined radiation dose behind the X-ray tube

The combined (total) radiation dose by scattered and leakage radiation was determined at 1 m and 2 m behind the X-ray tube. This position is typically occupied by the radiographer, whose distance to the X-ray tube is constrained by the length of the exposure cord.

**Fig 5:**
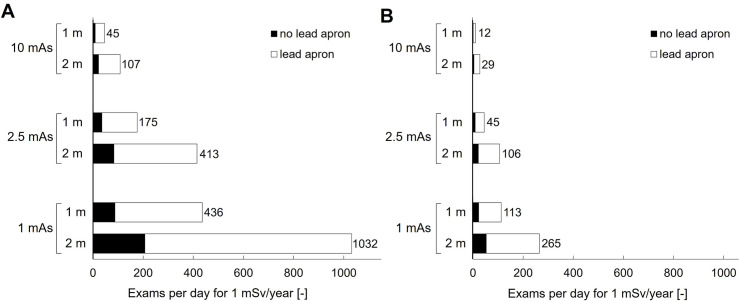
Number of exams that could be performed per day while keeping the combined radiation dose by scattered and leakage radiation at 1 and 2 m behind the X-ray tube below 1 mSv/year. A. Scan parameters were SSD 1.8 m and 90 kV. B. Scan parameters were SSD 1 m and 90 kV. The white bars illustrate the effect of wearing a lead apron and thyroid collar, using a correction factor (5x) as suggested by [20].

In Fig 5 the maximum number of exams is shown that can be performed while keeping the yearly total dose at this position below the international public radiation dose limit (1 mSv/year). The effect of various parameters is illustrated: Increasing SSD increased the maximum number of exams (Fig 5A vs. Fig 5B), e.g. at 1 m from the X-ray tube (at 90 kV, 2.5 mAs, no lead apron), the number of exams increased from 9 to 35, when the SSD was increased from 1 m to 1.8 m. Increasing mAs decreased the maximum number of exams. For example, at 1 m from the X-ray tube (90 kV, SSD 1.8 m, no lead apron), the number of exams decreased from 88 to 9 exams, when mAs was increased from 1 mAs to 10 mAs. Increasing the distance to the X-ray tube from 1 m to 2 m increased the maximum number of exams, e.g., the number of exams increased from 35 to 83, when the distance increased from 1 m to 2 m (90 kV, 2.5 mAs, SSD 1.8 m, no lead apron). Wearing a lead apron increased the maximum number of exams before exceeding the 1 mSv-limit. In the previous example (90 kV, 2.5 mAs, SSD 1.8 m), wearing a lead apron increased the maximum from 35 exams to 175 exams at 1 m from the X-ray tube and from 83 exams to 413 exams at 2 m from the X-ray tube.

## Discussion

UP X-ray devices are an ideal tool to facilitate CXR screening in community-based resource limited settings. However, radiation related health risks to the radiographers and others near the X-ray set-up must be considered, in line with international safety standards [13,16]. Our results indicated that for most CXR screening scenarios, it was possible to keep the total radiation dose by scattered and leakage radiation below the international limit to the public (1 mSv/year) for all individuals involved, if proper radiation safety precautions are taken (Fig 5). For radiographers, this is markedly lower than their international limit of 20 mSv/year.

The fundamental pillars of radiation safety are time, distance and shielding [13]. A combination of these pillars can be used to minimize an individual’s radiation dose. In community-based CXR screening, UP X-ray systems are frequently moved between locations. Therefore, it is often not practical or even impossible to transport heavy lead barriers to provide radiation shielding. As a result, for the public, i.e., non-radiographer program staff and people in waiting areas, maintaining a safe distance from the radiation source is the most effective method of protection: doubling the distance from the radiation source, reduces the dose by a factor four according to the inverse square law (2 x distance = 1/(2^2^) x dose [17]).

Our ‘bunny’ representation of the scattered radiation pattern provided minimum distances from the patient for various scenarios to help ensure individuals are positioned far enough to keep the dose below the 1 mSv/year public dose limit. For example, areas to the patient’s left and right, where the scattered radiation dose is lowest, are preferred for the administration or waiting area. However, even at these ‘low dose’ positions, for high volume screening projects a large distance (up to 5 m) would be needed, which might be a limitation for some CXR screening locations. Therefore, the time spent should be minimized and the non-radiographer program staff, who are present all year, should be carefully considered, e.g., by providing protective clothing, when necessary. The area behind the edges of the detector should be avoided, because here the scattered radiation dose was highest. At this location, the radiation dose could be even significantly higher if the primary X-ray beam is not consistently collimated (focused) on the patient, highlighting the importance of training for individuals operating UP X-ray devices. Also, additional radiation safety measures to prevent access to this area (e.g., safety ribbon) should be strongly considered.

The scattered radiation dose was also high in the direction back towards the X-ray tube. At this position, leakage radiation from the X-ray tube makes an additional contribution to the overall radiation dose. Therefore, this location should also be avoided by the public (including non-radiographer program staff). Unfortunately, the radiographers are constrained in the distance he/she can take from the X-ray tube, due to the exposure cord connected to the X-ray tube to operate the system. As a result, additional radiation protection measures are strongly advised to keep their radiation dose as low as possible. First, adequate means should be provided to ensure maximal distance can be created, e.g., a long cord for the X-ray exposure switch. From this perspective, using handheld X-ray systems as a true handheld (*i.e.,* with minimal distance between radiographer and X-ray tube) in high volume CXR screening programs will be suboptimal in terms of radiation safety. Secondly, as the IAEA advises, the use of shielding by wearing protective clothing (*i.e.,* lead aprons and thyroid collars) is strongly recommended when X-ray exams are performed outside of a dedicated X-ray facility [13], which is the intended use of UP devices [1]. Our results indicated that for most clinical scenarios lightweight lead gowns of 0.25 mm lead equivalent material provided sufficient radiation protection. Heavier lead gowns (e.g., 0.5 mm lead equivalent material) may not be required in these settings. Moreover, a potential disadvantage of heavier lead gowns, especially when used in hot and humid climates, may be decreased wearer compliance. Finally, an additional way to reduce the radiation exposure to the radiographers would be to reduce the time spent close to the UP X-ray system, e.g., by alternating work shifts or rotating roles (e.g., patient administration vs. radiographer) [13].

The radiation dose to both radiographers and the public is directly related to patient dose. In best clinical practice, the radiation dose to the patient should be as low as reasonably achievable (ALARA) without compromising diagnostic image quality [22]. This is achieved by optimizing X-ray scan parameters (*i.e.,* kV, mAs, SSD) for each individual patient. For UP X-ray systems performing CXR, the WHO/IAEA recommend a tube voltage capacity of at least 90 kV to obtain sufficient image contrast [1]. The appropriate mAs depends on patient size, with larger patients requiring higher mAs. For UP X-ray devices, radiographers must manually adjust the mAs-value, since these devices are not equipped with automatic exposure control. This fine-tuning is crucial because too high mAs poses an unnecessary radiation dose to patients without providing additional diagnostic benefit, while too low mAs leads to poor image quality and the necessity to repeat the X-ray exam, thereby doubling the radiation dose. The optimal mAs is also influenced by the distance between the X-ray tube and the patient. CXRs are typically performed at distances between 1–1.8 m, with longer distances generally preferred, but necessitating higher mAs to maintain image quality. Our results indicated that UP X-ray devices, if used according to ALARA, use slightly lower patient doses compared to conventional X-ray systems [23], thereby resulting in slightly lower levels of scattered radiation. However, it is not known whether this has a significant impact on image quality.

It is important to realize that the radiation dose of radiographers, but also the public, may well exceed the radiographer limit of 20 mSv/year if the above factors are not well understood or if they are not implemented properly. For example, in the highest dose scenario tested (90 kV, 10 mAs, SSD 1 m), a radiographer not wearing a lead apron and standing 1 m behind the X-ray tube, will exceed the 20 mSv/year limit at only 60 CXR exams per day, which is a fraction of the patient throughput in high volume screening programs. However, the use of 10 mAs is outside the normal clinical range for adult CXRs (so not in line with the ALARA-principle described above) and should generally not be used, unless exceptional circumstances require it. Furthermore, a high radiographer dose can be easily mitigated by following the basic radiation safety measures (time, distance, shielding). In the above example, if the radiographer would use more typical clinical mAs values (i.e., less than 2.5 mAs), wear a lead apron and take more distance (2 m), he/she could perform 2120 exams/day while staying below 20 mSv/year. From our experience, there are unfortunately large variations in local working habits, emphasizing the necessity of training and supervision of CXR screening staff. Importantly, when staff members are at risk of a radiation exposure above 1 mSv/year (the public dose limit), it is strongly advised to monitor staff with personal radiation dosimeters, so that prompt action can be taken when required [16,22]. However, access to reliable personal dosimetry services may be limited in some contexts, and is therefore important to address prior to implementation of an UP X-ray program [4].

In general, an important implication of this study is the necessity to ensure radiation safety training of all staff working with UP X-ray devices. Additionally, specific training for radiographers should be provided on X-ray imaging techniques, including the ALARA-principle and collimation of the X-ray beam to the body part being imaged (e.g., to the thorax) [22]. This is particularly important for CXR screening programs, where often non-radiographers or radiographers with limited training and expertise are involved, and there may be limited support networks in LMIC [24].

## Strengths and limitations

The strength of our study is that it was designed and performed by radiation protection experts (medical physicists), in close collaboration with a multidisciplinary team of radiographers, medical doctors and public health experts. The study was conducted in a controlled environment (i.e., phantom study), thereby creating a standardized set-up to test four UP X-ray devices under identical conditions. By using a generic approach to calculate the yearly radiation doses and effect of radiation protection measures, rather than comparing measurements of individual UP X-ray devices, these results can form a basis for general radiation safety guidelines.

One limitation of our study is that it was performed in a controlled experimental setting. Practical field settings could vary from situation to situation. To mitigate this limitation, several scenarios were evaluated. Future studies could focus on the correlation of the current results with personal radiation dosimeter readings of UP X-ray staff, including both radiographers and non-radiographer program staff.

Importantly, our study focused on the use of UP X-ray devices for CXR screening. The results and the radiation safety precautions are not universally applicable to all use cases of UP X-ray systems, because the amount of scattered and leakage radiation produced are dependent on 1. the body part being examined, 2. the scan parameters used and 3. the UP X-ray set-up (e.g., horizontal or vertical primary X-ray beam.

## Conclusion

Our study analyzed the radiation doses from UP X-ray devices when performing CXR exams on a thorax phantom in a controlled setting. It was found that they can be safely used, for example in community-based CXR screening, while keeping the radiation dose for both the radiographers and the public below international limits. However, for most clinical scenarios (depending on patient throughput and technical factors), this is only feasible when basic radiation safety measures are implemented such as ensuring adequate distance to the radiation source, and providing a lead apron and thyroid collar to the radiographers. These measures are crucial to prevent unnecessary health risks.

## Supporting information

S1 Table Description of ESD, scattered and leakage radiation. (PDF)

S2 Table Description of radiation dose units [22]. (PDF)

S3 Table Radiation dose limits [22]. (PDF)
