# Supplementary material for: Assessment of scattered and leakage radiation from ultra-portable X-ray systems in chest imaging: An independent study
Source: PLOS Glob Public Health. 2025 Jan 24;5(1):e0003986. doi: 10.1371/journal.pgph.0003986 (PMC11761074; doi:10.1371/journal.pgph.0003986)
Supplement: S1 Table — (PDF) [file pgph.0003986.s001.pdf]

S1 Table. Description of ESD, scattered and leakage radiation.

Parameter	Unit*	Description
ESD	mGy or mSv [J/kg]	X-ray radiation dose from the primary X-ray beam at the level of the patient’s skin (where the X-ray beam enters the patient). It can be used as an indirect measure of X-ray tube output
Scattered radiation	mGy or mSv [J/kg]	X-ray radiation that is scattered from the patient to its surroundings, upon interaction of the primary X-ray beam with the patient
Leakage radiation**	mGy or mSv[J/kg]	X-ray radiation that emanates from the X-ray tube’s protective housing in other directions than the primary X-ray beam

* Conversion from Gy to Sv can be performed by a correction factor of 1.4, as retrieved from [18].

** Please note there is no leakage radiation when the X-ray tube is not producing a primary X-ray beam (beam-on time), because there is no radioactive material.
