# Supplementary material for: Assessment of scattered and leakage radiation from ultra-portable X-ray systems in chest imaging: An independent study
Source: PLOS Glob Public Health. 2025 Jan 24;5(1):e0003986. doi: 10.1371/journal.pgph.0003986 (PMC11761074; doi:10.1371/journal.pgph.0003986)
Supplement: S2 Table — (PDF) [file pgph.0003986.s002.pdf]

S2 Table. Description of radiation dose units [22].

Parameter	Unit	Description
Absorbed dose	mGy [J/kg]	Absorbed energy from X-ray photons by a specific mass
Effective dose	mSv [J/kg]	Absorbed energy from X-ray photons by a specific mass, taking into consideration the difference in biological effect on different tissue types

Conversion from Gy to Sv was performed by a correction factor of 1.4, as retrieved from [18].
