# Supplementary material for: Assessment of scattered and leakage radiation from ultra-portable X-ray systems in chest imaging: An independent study
Source: PLOS Glob Public Health. 2025 Jan 24;5(1):e0003986. doi: 10.1371/journal.pgph.0003986 (PMC11761074; doi:10.1371/journal.pgph.0003986)
Supplement: S3 Table — (PDF) [file pgph.0003986.s003.pdf]

S3 Table. Radiation dose limits [22].

		Effective dose
**Occupational**	**> 18 years**	20 mSv/year averaged over 5 consecutive years, or 50 mSv/year in any single year
	**16 - 18 years**	6 mSv/year
	**pregnant**	See ‘Public’
**Public**		1 mSv/year averaged over 5 consecutive years
